# Post-translational modifications of collagen type I in osteogenesis imperfecta: Systematic review and meta-analysis^☆^

**DOI:** 10.1016/j.bonr.2025.101894

**Published:** 2025-12-12

**Authors:** Priyesh Patel, Sirion Aksornthong, Svetlana V. Komarova

**Affiliations:** a Faculty of Dental Medicine and Oral Health Sciences, McGill University, Montréal, Canada; b Shriners Hospital for Children – Canada, Montréal, Canada; c Department of Experimental Surgery, Faculty of Medicine and Health Sciences, McGill University, Montreal, QC, Canada; d Department of Biomedical Engineering, Faculty of Engineering, University of Alberta, Edmonton, Alberta, Canada

**Keywords:** Osteogenesis imperfecta, Type 1 collagen, Post-translational modification, Hydroxylysine, Hydroxyproline, Dentine, Rare disease, meta-analysis

## Abstract

Osteogenesis imperfecta (OI) is a rare genetic disorder most often caused by mutations in genes that encode collagen type I. OI collagen-I differs from healthy collagen-I due to the underlying mutation and altered post-translational modifications (PTMs). The objective of this study was to use knowledge synthesis to quantify the levels of selected PTMs, hydroxylysine (HYL), hydroxyproline (HYP) and glycosylation (GLY) in OI collagen-I. A systematic search in Medline, Ovid and Web of Science, identified 701 studies reporting on PTM outcomes for OI patients with collagen-I mutation. We excluded animal studies, and reports for OI patients with mutations other than in collagen-I. After screening, we included 36 qualitative studies and 25 quantitative studies for meta-analysis. All qualitative studies reported that OI collagen-I was overmodified. Meta-analysis of studies with quantitative data was performed using normalized mean difference as a study-level effect size and a random-effects model with the Hunter and Smith with sample size correction. The hydroxylysine dataset included 150 patients across 20 studies and had an effect size of 0.33 (confidence interval (CI) 0.21, 0.45). HYL levels were higher in bone-derived collagen-I than in fibroblast- or dentine-derived. The hydroxyproline dataset included 141 patients across 17 studies and had an effect size of 0.00 (CI: −0.02, 0.02). The glycosylation dataset included 17 patients across 5 studies and had an effect size of 0.55 (CI: 0.38, 0.71). Patients with the most severe form of OI (type 2) had the highest levels of collagen-I HYL and GLY. Our study provides new insights into collagen-I pathophysiology in OI, generating new hypotheses regarding the role of PTM in mediating disease presentation in different tissues and overall severity.

## Highlights

• Mutations and biochemical changes alter collagen-I in osteogenesis imperfecta.
• Post-translational modifications analyzed across 66 studies, 269 patients.
• Hydroxylysine and glycosylation increased; hydroxyproline unchanged in OI collagen-I.
• Collagen-I more modified in bone than in skin/dentine, and in severe OI cases.
• Altered collagen may explain tissue-specific disease patterns and severity.

## Graphical abstract

**Figure:**
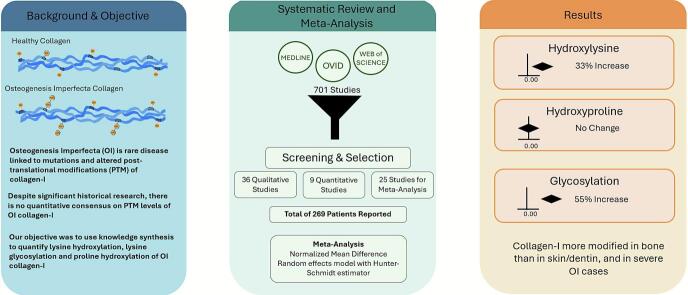

## 1 Introduction

Osteogenesis imperfecta (OI) is a rare metabolic bone disorder affecting 1 in every 15,000 births. Most OI cases (85 %) are caused by a mutation in one of the two genes that encode for type 1 collagen, COL1A1 and COL1A2 (Rauch and Glorieux, 2004; Tournis and Dede, 2018; Nijhuis et al., 2019; Tauer et al., 2019). Type 1 Collagen (collagen-I) is the most prevalent protein in bone tissue. OI is synonymous with brittle bone disease, as the main characteristic of OI is bone fragility. Additionally, phenotypic presentation includes skeletal deformities, scoliosis, low bone mineral density, and dentinogenesis imperfecta (Tournis and Dede, 2018). Presentation of OI with collagen-I mutations is classified under four categories, OI type I-IV, of which OI type I is mild; OI type II is the most severe and associated with perinatal or neonatal lethal cases, OI type III is severe and OI type IV is moderate-severe (Van Dijk et al., 2010).

The essential characteristic of collagen-I is that it is made of 2 COL1A1 chains and 1 COL1A2 chain interwoven into a triple helix (Buss et al., 2022). Each chain has a repetitive amino acid sequence G-X-Y, where G is glycine, X is likely proline, and Y is likely hydroxyproline. Collagen-I undergoes several post-translational modifications (PTM) during the triple helix formation. Both proline and lysine are modified (Gjaltema and Bank RA, 2017; Naomi et al., 2021). Proline is hydroxylated to form hydroxyproline (HYP); the reaction is catalyzed primarily by prolyl 4-hydroxylase (P4H). The hydroxylation by P4H causes a conformational change, which helps with the assembly of the triple helix (Chow et al., 2018). Lysyl hydroxylase converts lysine to 5-hydroxylysine (HYL). Glycosyltransferase 25 domain-containing 1 catalyzes the addition of galactose to HYL to form galactosyl hydroxylysine (GHYL). Glycosyltransferase 25 domain containing 2 catalyzes the addition of glucose to GHYL to form glucosyl galactosyl hydroxylysine (GGHYL) (De Giorgi et al., 2021). OI collagen-I differs from healthy collagen-I due to the underlying mutation and the alterations in PTM levels (Lehmann et al., 1995). There is currently no quantitative consensus on the PTM levels in OI.

We performed a systematic review and meta-analysis on OI collagen-I post-translational modification. The primary objective was to quantify HYL, HYP, and glycosylation levels in OI collagen-I compared to healthy controls. The secondary objective was to examine the association between PTM levels and disease severity.

## 2 Materials and methods

### 2.1 Information sources, search strategy, eligibility criteria, and screening

This study complies with the Preferred Reporting Items for Systematic Review and Meta-Analysis (PRISMA) statement (**Supplementary Table 1**) (Page et al., 2021). The search strategy used combined keywords and Medical Subject Headings (MESH) terms related to OI and terms related to collagen post-translational modification (**Supplementary Methods 1**). The search was performed in Medline, Embase, and Web of Science in January 2023 and updated in January 2025. The systematic review was not registered in PROSPERO. The title/abstract screening was performed independently by two reviewers (PP and SA) using the Rayyan Systematic Review Screening Software (Ouzzani et al., 2016). Disagreements between screeners were resolved by discussion. The full text screening was performed by PP with the inclusion criteria of studies experimentally assessing collagen-I PTM in samples from OI patients. The included studies were grouped as *i)* studies containing quantitative assessment of lysine hydroxylation, proline hydroxylation, and hydroxylysine glycosylation suitable for the meta-analysis; ii) studies reporting quantitative data on collagen-I PTM that were incompatible with meta-analysis due to unit/method differences; iii) studies containing qualitative data only. The exclusion criteria were studies describing OI types that are not due to collagen-I mutations, animal studies, conference abstracts, reviews, and editorial commentary. The full list of included studies is in the **Supplementary bibliography**.

### 2.2 Data extraction

Publication information and patient parameters were extracted from studies in all three groups (meta-analysis, quantitative and qualitative). It includes authors, publication year, data type presentation (individual or aggregate), number of patients/controls, patient OI type (collagen-I mutation was noted), age and sex of participants. Some studies did not explicitly state the OI type of the patient; however, the disease severity could be determined by the description of the patient (such as perinatal lethal OI type II). For qualitative data, the presence of α,α-dipyridyl was noted. For the meta-analysis, we extracted the PTM levels with variance measures in patient and control samples, the collagen processing technique, the units of measurement for PTM (**Supplementary Table 2**), and the origin of the collagen samples (bone, fibroblast, or dentine).

### 2.3 Data conversion

The common outcome units for HYL and HYP were percentage hydroxylysine and percentage hydroxyproline, expressed as $\mathrm{Percentage Hydroxylated}=\frac{\mathrm{hydroxylated amino acid}}{\mathrm{hydroxylated amino acid}+\mathrm{amino acid}}$. When the outcome was presented as amino acid count, this formula was used. When the outcome was presented as a ratio ($\frac{\mathrm{Hyl}}{\mathrm{lys}}$ or $\frac{\mathrm{Hpr}}{\mathrm{pro}}$), it was converted to percentage with $\mathrm{Percentage Hydroxylated}=\frac{\mathrm{ratio}}{\mathrm{ratio}+1}\;$. When the amino acid count was given by cyanogen bromide peptide, the amino acid counts for each peptide were added up, and then percentage was calculated. When the percentage hydroxylation was given per chain of collagen, a weighted average was calculated (66 % for A1 chains and 33 % for A2 chains). The ratio of $\frac{\mathrm{HYL}}{\mathrm{HPR}}$ was another unit of interest and it was calculated using the amino acid counts, except when given directly. For the glycosylation outcomes, the common outcome presentation was as the ratio of diglycosylated/monoglycosylated (GGHYL/GHYL) hydroxylysine.

### 2.4 Study level outcomes

The selected effect size was the normalized mean difference, calculated as $\theta_{i}=\frac{\theta_{i}^{p}-\theta_{i}^{c}}{\theta_{i}^{c}}$, where $\theta_{i}^{p},\;\theta_{i}^{c}$ were means for the patient and control populations in study *i (*Mikolajewicz and Komarova, 2019*)*. For studies reporting data for fibroblast and bone samples, the effect size was calculated for these samples independently and then the average of both samples was used for meta-analysis. For studies reporting aggregated data by OI type, the effect size was calculated for each group, followed by calculating a weighted average based on the number of patients in each group.

To estimate the standard errors, we used the following considerations. For one study reporting aggregate data, we use the reported standard deviation (SD), $\mathrm{sd}\left(\theta_{i}\right)$ for patient and control populations. For studies that reported individual patient data (IPD) for 4 or more patients or controls per outcome (4 of 17 studies for HYL dataset, 2 of 14 studies for HYP dataset, and 1 of 5 studies for Gly dataset) the standard deviations for patients and controls were calculated as $\mathrm{sd}\left(\theta_{i}\right)=\sqrt{\frac{\sum_{i=1}^{n}{\left(x_{i}-\bar{x}\right)}^{2}}{n-1}}$, where $x_{i}$ represents individual datapoints, and $\bar{x}$ is the mean of individual datapoints. To estimate the SD for samples in studies with fewer than 4 patients or controls, first, we calculated the combined SD for all data points in small-sized studies, treating data for patients and controls separately. Then, the combined SD for patients was imputed to patient or patient groups in each study with 4 or less patients, while the combined standard deviation for controls was assigned to control or control groups in each study with 4 or less controls (Furukawa et al., 2006; Seo et al., 2021). The study-level standard error was calculated as $\mathrm{se}\left(\theta_{i}\right)=\sqrt{\frac{{\left(\frac{\mathrm{sd}\left(\theta_{i}^{c}\right)}{\theta_{i}^{c}}\right)}^{2}}{n_{i}^{c}}+\frac{{\left(\frac{\mathrm{sd}\left(\theta_{i}^{p}\right)}{\theta_{i}^{p}}\right)}^{2}}{n_{i}^{p}}\;}$, where $\mathrm{sd}\left(\theta_{i}^{c}\right)\;\mathrm{and}\;\mathrm{sd}\left(\theta_{i}^{p}\right)\;$ are the SD from the pooled control and patient data, $n_{i}^{c}\mathrm{and}\;n_{i}^{p}$ is the number controls and patients within the study.

### 2.5 Meta-analysis

Meta-analysis was performed using a random effects model (Riley et al., 2011). The global effect size ($\hat{\theta })$ and its standard error was calculated using the Hunter-Schmidt estimator with small sample size correction (Brannick et al., 2019). Confidence intervals (CI) were calculated as $95\%\mathrm{CI}=\hat{\theta }\pm Z_{\left(1-\frac{\alpha }{2}\right)}\times \mathrm{SE}\left(\hat{\theta }\right)=\hat{\theta }\pm 1.96\times \mathrm{SE}\left(\hat{\theta }\right)$. Heterogeneity analysis was performed, and the percentage of variation across studies due to heterogeneity, I^2^, and between-study variance, τau^2^, were reported. Assumption of the methodologies described is presented in **Supplementary Method 3**.

To evaluate the covariate effect, the individual effect size was calculated by comparing the individual patient value with the average control value. Then, the individual patient effect sizes were combined by their covariates (Lambert et al., 2002). Only studies in which the analyzed covariates were explicitly reported were included in this analysis.

### 2.6 Assessment of bias

Studies selected for meta-analysis were evaluated for bias using a quality assessment tool that includes questions to assess case studies. The assessment consists of 10 questions, with a maximum obtainable score of 11 points (**Supplementary Methods 2**). A funnel plot was made for each outcome to visualize study-level reporting bias (Viechtbauer and Cheung, 2010).

### 2.7 Software

EndNote 20 and Rayyan were used for reference management (Ouzzani et al., 2016). Webplot digitizer was used for data extraction from figures. Microsoft Excel version 2507 build 16.0.19029 was used for data management, effect size and standard error calculations. Python with the matplotlib package was used to make some figures (Hunter, 2007). R version 4.2.2 was used in Visual Studio Code version 1.103.1 with the metafor package for meta-analysis (forest plot, heterogeneity analysis, funnel plot), and the tidyplots package for figure preparation (Engler, 2025; Viechtbauer, 2010).

## 3 Results

**Fig. 1:**
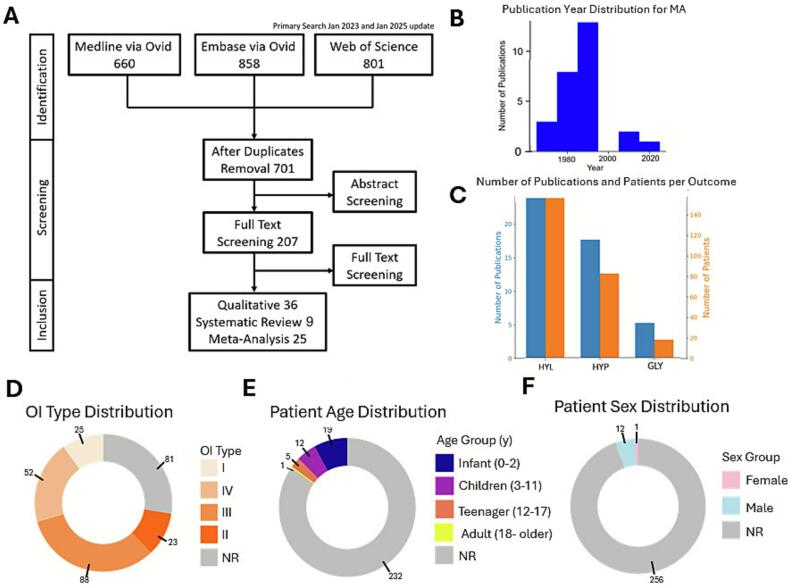
**Systematic review and meta-analysis information flow.** (A) The PRISMA diagram indicates the flow of assessment in each step of the process. (B) The publication year distribution for all included studies. (C) Bar plots for number of publications (blue, left y-axis) and patients (orange, right y-axis) for meta-analysis of collagen modifications for hydroxylysine (HYL), hydroxyproline (HYP) and glycosylation (GLY). (D, E, F) Pie charts visualizing reporting of patient parameters including OI type (D), age (E), and sex (F). NR: not reported.

A systematic search was conducted across three databases: Medline (*n* = 660), Embase (*n* = 858), and Web of Science (*n* = 801), which after duplicate removal, identified 701 unique studies. These studies were screened by title and abstract, and 207 were selected for full-text evaluation (Fig. 1A). After meeting the inclusion criteria of describing PTM of collagen-I in OI patients, 70 studies primarily published in the early to late 90s (Fig. 1B) were included in the systematic review. Of these, 36 studies only provided the qualitative data, 9 reported quantitative outcomes which could not be combined with other studies, and 25 studies were included for meta-analysis (complete reference lists are in **Supplemental bibliography**). The meta-analysis included studies assessing PTMs in bone, fibroblast and dentine-derived collagen-I, including 17 studies of hydroxylysine (HYL) levels in 88 OI patients, 14 studies of hydroxyproline (HYP) levels in 79 patients, and 5 studies of glycosylation ratios (GLY) in 17 patients (Fig. 1C). Additionally, three studies reported PTMs in dentine collagen-I in 62 patients. A total of 269 patients were described. The OI type distribution was balanced across all four types and for 69 patients, the OI type was not reported (NR) (Fig. 1D). The age was not reported for the majority of patients. When reported, it was mainly for the pediatric population (Fig. 1E). The patient sex was only reported for 13 of 269 patients (Fig. 1F).

The qualitative dataset comprised 36 studies, which described 58 patients (Table 1). These studies report overmodified collagen-I by isolating the collagen-I from OI patients and using gel electrophoresis to compare the thickness of the collagen-I band to that of the control, where the thicker bands indicate overmodified collagen (Barsh and Byers, 1981). In addition, some studies used the iron chelator α,α-dipyridyl to inhibit lysyl oxidase, which reduced the band thickness to the control levels, confirming that the increase in thickness is related to an increase in PTM levels (Ikeda et al., 1992). All studies in the qualitative dataset reported overmodified collagen-I in OI patients (Table 1).

**Table 1:** **Overview of studies included in qualitative analysis.** Shown are the number of patients (NP) and controls (NC), OI type or reported severity, patients' age (in years, y), and sex (male (M), females (F), α,α-dipyridyl compound used as control for post-translational modification, Overmodified in OI based information reporting. Abbreviation: NR, not reported.

Author and title	NP	NC	OI type	Age (y)	Sex	α,α- Dipyridyl	Overmodified in OI
Barsh, 1981	1	1	II	0.5	NR	Yes	Yes
Sippola, 1984	1	1	Mild	5	M	Yes	Yes
De Vries, 1986	1	1	NR	59	F	Yes	Yes
Wenstrup, 1986	1	1	III	8	M	Yes	Yes
Wenstrup, 1986	1	1	IV	1	M	Yes	Yes
Vogel, 1987	1	1	II	2	M	Yes	Yes
Bateman, 1987	1	1	II	Perinatal lethal	F	Yes	Yes
Bateman, 1987	1	1	II	NR	F	No	Yes
Willing, 1988	1	1	II	Perinatal lethal	NR	Yes	Yes
Byers, 1988	1	1	II	Perinatal lethal	NR	Yes	Yes
Tenni, 1988	3	1	III	2,9,10	M	Yes	Yes
Bateman, 1988	1	1	III	NR	NR	Yes	Yes
Superti-Furga, 1988	1	1	III	NR	M	No	Yes
Wenstrup, 1988	1	1	I	42	F	Yes	Yes
Marini, 1989	1	1	IV	4	F	Yes	Yes
Constantinos, 1989	1	1	II	Perinatal lethal	F	Yes	Yes
Baldwin, 1989	1	1	II	Perinatal lethal	F	Yes	Yes
Starman, 1989	3	1	I	21	F	Yes	Yes
Wallis, 1990	1	1	II	NR	NR	Yes	Yes
Grange, 1990	1	1	II	Perinatal lethal	F	Yes	Yes
Valli, R. 1990	2	1	I	4,5	M,F	Yes	Yes
Bateman, 1991	1	1	IV	NR	F	No	Yes
Tenni, 1991	1	1	I	2	M	Yes	Yes
Hawkins, 1991	1	1	II	NR	NR	No	Yes
Nicholls, 1991	1	1	III	NR	NR	No	Yes
Wallis, 1992	1	1	II	Perinatal lethal	M	No	Yes
Bateman, 1992	1	1	II	Perinatal lethal	NR	No	Yes
Fertala, 1993	2	1	II	Perinatal lethal	NR	No	Yes
Valli, 1993	1	1	II	18 months	F	No	Yes
Marini, 1993	2	1	IV	4,8	F	Yes	Yes
Lightfoot, 1994	1	1	IV	2	M	No	Yes
Sarafova, 1998	3	1	IV	9,5,15	M,F	Yes	Yes
Pace, 2001	1	1	I-IV	NR	NR	No	Yes
Pace, 2001	1	1	I	NR	M	No	Yes
Pace, 2002	1	1	II	Perinatal lethal	F	No	Yes
Cabral, 2003	4	1	II & NR	NR	NR	No	Yes

Nine quantitative studies describing 51 patients were not included in the meta-analysis (Table 2). Six of 9 studies reported outcomes related to glycosylation and 5 studies reported outcomes related to lysine hydroxylation. Glycosylation outcomes reported were percentage glycosylation; however, it was unclear what this percentage specifically designates (diglycosylated/all lysine residues, diglycosylated/hydroxylated lysine residues, diglycosylated/monoglycosylated + hydroxylated lysine residues), preventing their inclusion in meta-analysis. The two studies by Taga and colleagues (Taga et al., 2013; Takeyari et al., 2021) demonstrated site-specific hydroxylation and glycosylation of lysine, indicating that each site had its own distribution of hydroxylation and glycosylation levels in OI patients. These studies show that there is significant heterogeneity with regard to PTM levels (LYS, HYL, GHYL and GGHYL) at each site. All the studies with quantitative data on glycosylation reported a higher glycosylation levels in OI collagen-I compared to controls.

**Table 2:** **Overview of studies containing quantitative data excluded from the meta-analysis**. Shown are number of patients (NP) and controls (NC), OI type, patients' age (in years, y), and sex (male (M), females (F)), reported quantitative data for outcomes (collagen modification of hydroxylysine (HYL), hydroxyproline (HYP), glycosylation (GLY)), Units represents the units that was used for data reporting. Abbreviation: NR, not reported.

Author and year	NP	NC	OI Type	Age (y)	Sex	Outcome	Units
Trelstad, 1977	1	1	II	Perinatal lethal	F	GLY	Percentage of glucose, galactose
Kirsch, 1981	1	1	II	0.25	M	GLY	Relative amount of glycosylated hydroxylysine and Percentage of the sum of glycosylated hydroxylated
Bateman, 1984	7	2	II	Perinatal lethal	NR	HYL, GLY	Percentage Hydroxylation per Chain (missing data)
Bateman, 1987	1	1	II	Perinatal lethal	F	HYL	HYL residues per CNBR peptide of A1 Chain
Kirsch, 1987	4	2	II	Perinatal lethal	NR	GLY	Percentage Glycosylated
Tajima, 1994	1	3	IV	12	M	GLY	Percentage Glycosylated
Bank, 2000	28	61	III,IV	NR	NR	HYL	HYL/triple helix
Taga, 2012	2	1	II & NR	NR	NR	HYL, GLY	site specific hydroxylation and glycosylation levels
Takeyari, 2020	6	1	I & III	NR	NR	HYL,GLY	site specific hydroxylation and glycosylation levels

From 25 quantitative studies included in meta-analysis, 13 were case reports with 1 patient each, 4 were case series, with 2–4 patients per study, and 8 were cohort studies with 13–59 patients per study (Table 3). The number of control samples was generally similar to that of patients, except for three studies, which reported 3 to 7 control samples for 22–30 patient samples (Brenner et al., 1989; Brenner et al., 1993; Brenner et al., 1988). The study level effects were calculated as a normalized mean difference between PTM levels in patient and control samples, with values higher than 0 indicating higher levels in OI patients. For studies reporting aggregate data or IPD for more than 4 patients and controls, we used the reported or calculated mean and SD to estimate the study level effect size and its variance. For studies reporting 1–3 patient or control samples, we calculated the global variance for both patients and controls by combining the SD from all the studies reporting the same PTM outcome. The global variance was then imputed to the patients and controls in relevant studies, followed by calculation of the effect size and its variance (standard error). For statistical independence, when data for the same outcome assessed in different samples of the same patient were reported, the effect size was calculated for each sample independently and then averaged.

**Table 3:** **Overview of studies included in meta-analysis.** Shown are number of patients (NP) and controls (NC), OI type, patients' age (in years, y), and sex (male (M), females (F)), reported outcomes (collagen modification of hydroxylysine (HYL), hydroxyproline (HYP), ratio of hydroxylysine to hydroxyproline (HYL/HYP), glycosylation (GLY)), and quality scores (QS; out of 11 maximum). Abbreviation: NR, not reported; PL, perinatal lethal; B, bone; F, fibroblast; D, dentine.

Author and year	NP	NC	OI type	Age (y)	Sex	Sample origin	Outcomes	Quality score (n/11)
Bleckmann, 1971	1	1	NR	3	M	B	HYL, HYP, HYL/HYP	5
Eastoe, 1973	1	1	II	PL	M	B, D	HYL, HYP, HYL/HYP	5
Meigel, 1975	1	1	NR	NR	NR	F	HYP	4
Trelstad, 1977	1	1	II	PL	F	B, F	HYL	7
Takagi, 1980	2	3	Tarda and cogenita	4, 13	NR	D	HYL	4.5
Kirsch, 1981	1	2	II	PL	M	B, F	HYL, HYP, HYL/HYP	8
Herbage, 1982	4	1	Moderate/severe and severe	Range 4 to 16 year	M,F	B, F	HYL, HYL/HYP	6.5
Kirsch, 1983	1	1	II	PL	NR	B	HYL, HYP, HYL/HYP	6
Cetta, 1983	13	12	Mild and severe	NR	NR	F	GLY	5.5
Steinmann, 1984	1	3	II	NR	M	F	HYL, HYP, GLY	8
Deak, 1985	1	1	NR	NR	NR	F	HYL, HYP, HYL/HYP	3
Stoss, 1986	1	1	III	PL	M	B	HYL, HYP, HYL/HYP	8
Kirsch, 1987	23	10	II	NR	NR	F	HYL	6.5
Tenni, 1988	1	2	I to IV	0 to 20	NR	F	HYL, GLY	9
Brenner, 1988	29	6	II	5	M	B	HYL/HYP	2
Maroteaux, 1988	3	3	I to IV	NR	NR	F	HYL, HYP, GLY	3
Gage, 1988	59	33	NR	3 months, 15, 27	NR	D	HYL, HYP	5
Rao, 1989	1	1	NR	NR	NR	F	HYL,HYP	7.5
Brenner, 1989	30	7	II	NR	M	B	HYL/HYP	8
Brenner, 1993	22	3	I to IV	NR	NR	B	HYL/HYP	4
Tajima, 1994.	1	1	III & IV	NR	NR	F	HYL, HYP, GLY	8
Lehmann, 1995	39	14	IV	12	M	F	HYL, HYP	7
Bank, 2000	28	51	I to IV	NR	NR	B	HYP	6
Barnes, 2006	1	1	III	NR	NR	F	HYL	7
Makareeva, 2018	4	1	NR	NR	NR	F	HYL	4.5

**Fig. 2:**
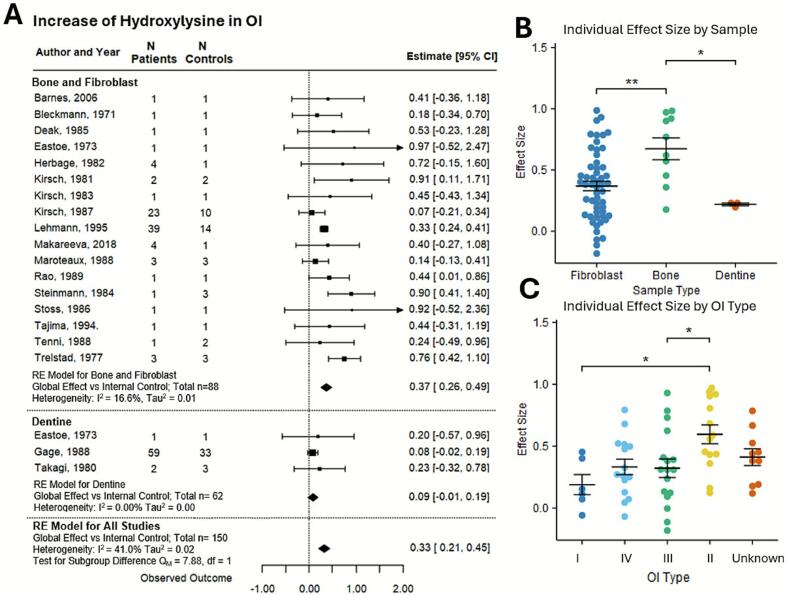
**HYL percentage in collagen of OI patients compared to controls.** (A) Forest plot of percentage difference in HYL in OI collagen, positive values indicate higher levels in patients. Squares with lines depict the study-level effect, normalized mean difference with 95 % Cls; the size of the square is proportional to the study weight. The diamond represents the global effect size. Heterogeneity statistics I^2^ and τau^2^ are reported. (B) HYL percentage in individual patient data sampled from fibroblasts, bone and dentine. (C) HYL percentage in individual patient data separated by OI type, ordered from least to most severe (I,IV,III,II). Unknown are samples in which OI type is unclear. B, C: shown are individual data points and their mean ± SEM; *p < 0.05 and ** *p* < 0.01 indicate significant differences by 1-way ANOVA with Tukey's post hoc test.

A meta-analysis of the change in HYL in OI collagen-I demonstrated a significant overall increase in hydroxylysine in OI collagen, with a global effect size of 0.33 [95 % CI: 0.21, 0.45] (Fig. 2A). Heterogeneity was minimal, with τau^2^ estimated at 0.02 and I^2^ at 41 %. When considering subgroup analysis, the bone and fibroblast had an effect size of 0.37 [95 % CI: 0.26, 0.49], and the dentine subgroup had an effect of 0.09 [95 % CI:- 0.01, 0.19]; the difference was significant, with a Q_M_ value of 7.88. To evaluate the covariate effect on PTM levels, we analyzed the individual patient data for which the covariate was explicitly reported. Bone-derived collagen-I demonstrated significantly higher HYL levels compared to fibroblast-derived and dentine-derived collagen-I (Fig. 2B). Since only individual patient data were used for this analysis, there were only 3 individual patients represented from the 62 total patients in the dentine group. We also identified three publications in which data for HYL in fibroblast-derived and bone-derived collagen-I were reported for the same individuals. While in OI patients, the HYL levels were consistently higher in bone-derived collagen-I compared to fibroblasts-derived, in control individuals HYL levels were lower in bone-derived samples (**Supplementary Fig. S1**). Next, the IPD for patients with reported OI type was analyzed for the association of HYL levels with OI severity. The most severe OI type II exhibited the highest HYL levels, while the least severe OI type I showed the lowest HYL levels (Fig. 2C). OI type II collagen-I HYL was significantly different from OI type I and type III. It is important to note that the bone-derived collagen-I was more commonly obtained from OI type II patients; therefore, it is difficult to interpret whether the OI severity or the tissue origin of OI collagen is the main driver of high HYL levels.

**Fig. 3:**
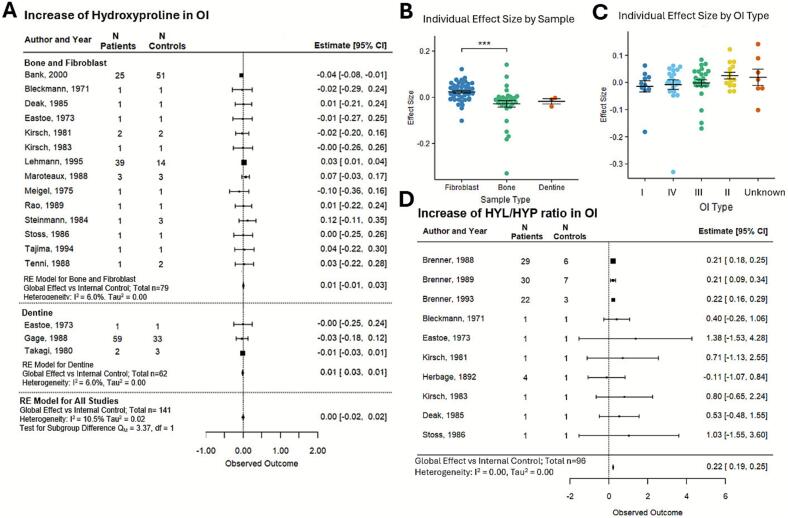
**HYP percentage in collagen of OI patients compared to control.** (A) Forest plot of the percentage difference in HYP in OI collagen compared to the control, with positive values indicating higher levels in patients. (B) HYP percentage in individual patient data sampled from fibroblasts, bone and dentine. (C) HYP percentage in individual patient data separated by OI type, ordered from least to most severe (I,IV,III,II). Unknown are samples in which OI type is unclear. (D) Forest plot of percentage difference in the ratio of HLY to HYP in OI compared to control collagen. For A and D, squares with lines depict the study level effect, normalized mean difference with 95 % Cls; the size of the square is proportional to the study weight. The diamond represents the global effect size. Heterogeneity statistics I^2^ and τau^2^ are reported. B, C: shown are individual data points and their mean ± SEM; ***p < 0.0001 indicate significant differences by 1-way ANOVA with Tukey's post hoc test.

A meta-analysis of the change in HYP in OI collagen-I demonstrated similar levels of HYP in OI and control collagen, with a global effect size of 0.00 [95 % CI: −0.02, 0.02] (Fig. 3A)**.** Heterogeneity was minimal, with τau^2^ estimated at 0.02 and I^2^ at 10.5 %. The OI collagen-I HYP levels were similar in bone- and dentine-samples, and higher in fibroblast-derived samples; nevertheless, overall effect sizes remained very small (Fig. 3B). HYP levels were not associated with OI severity (Fig. 3C). The HYL to HYP amino acid ratio was directly reported in a number of studies (Table 3) or it was calculated from the studies reporting both HYL and HYP. The HYL/HYP ratio was significantly higher in OI samples compared to control, with a global effect size of 0.25 [95 % CI: 0.23, 0.28] (Fig. 3D). Heterogeneity was minimal with a τau^2^ of 0.00 and an I^2^ of 0.0. These data are consistent with higher levels of HYL and similar levels of HYP in OI collagen-I compared to control.

**Fig. 4:**
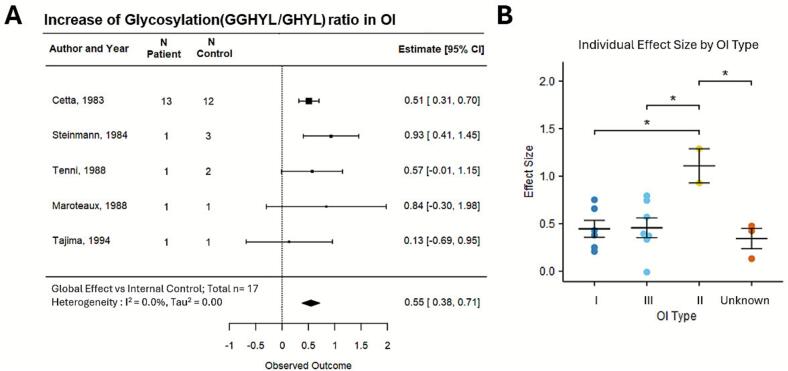
**Change in glycosylation ratio in collagen of OI patients compared to controls.** (A) Forest plot of the percentage difference in glycosylation ratio (diglycosylated/monoglycosylated) in OI collagen compared to the control, with positive values indicating higher levels in patients. Squares with lines depict the study-level effect, normalized mean difference with 95 % Cls; the size of the square is proportional to the study weight. The diamond represents the global effect size. Heterogeneity statistics I^2^ and τau^2^ are reported. (B) Glycosylation percentage in individual patient data separated by OI type, ordered from least to most severe (I,IV,III,II). Unknown are samples in which OI type is unclear. Shown are individual data points and their mean ± SEM; *p < 0.05 indicates significant differences by 1-way ANOVA with Tukey's post hoc test.

A meta-analysis of the GLY ratio in OI collagen-I demonstrated a significantly higher GLY ratio in OI samples compared to controls, with a global effect size of 0.55 [95 % CI: 0.38, 0.71] (Fig. 4A). The heterogeneity was zero with a τau^2^ of 0.00, and I^2^ of 0.0 %. GLY ratio was significantly higher in the most severe OI type II compared to OI type I and III (Fig. 4B).

To analyze the publication bias, we generated the funnel plots for HYL, HYP and GLY datasets. The studies tended to cluster around the global effect size; the plots were generally symmetrical, although some asymmetry can be noted for the HYL dataset (**Supplementary Fig. S2A—C**). We also examined whether there is an association between the reported effect size and the quality score of the study. We observed no evidence of over-reporting in lower quality studies (**Supplementary Fig. S2D—F**).

## 4 Discussion

This systematic review and meta-analysis compiled and quantified all available biological data on the post-translational modification of collagen-I in individuals with OI due to mutations in the collagen-I gene. Using the knowledge synthesis methodology allowed us to combine the data for 269 patients, a substantial sample size for a rare disease. We identified significantly higher levels of HYL and GLY in OI patients compared to control individuals; however, the HYP level remained unchanged. We have found that patients with the most severe form of OI (type II) had the highest levels of HYL and GLY, suggesting a potential role of high collagen-I PTM in mediating disease severity. We also identified that collagen-I derived from bone samples was more susceptible to over-modification, compared to fibroblast-derived and dentine-derived collagen, which is consistent with the knowledge that bone physiology is strongly affected in OI, even though collagen-I is expressed in many tissues. Taken together, our study provides new insights into collagen-I physiology and pathophysiology in OI. It generates new hypotheses regarding the role of PTM in mediating disease presentation in different tissues and overall severity.

We demonstrated that OI collagen-I is significantly post-translationally overmodified, specifically on its lysine residues, while proline residues remain unaffected. The PTM proteins are known to only bind to the individual chains and not to the assembled triple helix (Brownell and Veis, 1976). In the individual chain, hydroxylation of prolines at the Y position of the G-X-Y residues motif is specifically mediated by P4H and is a prerequisite for the triple helix formation (Salo et al., 2024). Thus, in control collagen-I, all proline residues in the Y position are likely hydroxylated, and proline hydroxylation in other positions is harder to achieve, suggesting a saturation for proline hydroxylation, which is maintained in OI collagen. In contrast, lysine hydroxylation by LH occurs on any lysine residue, and hydroxylated lysine can be further glycosylated. Over-modification of lysine supports the notion that OI collagen-I spends longer time in unfolded state (Raghunath et al., 1994). In keeping, it was shown that delaying collagen-I folding by increasing temperature leads to increased HYL levels while HYP levels remained the same (Torre-Blanco et al., 1992; Fujii et al., 2022). The estimates for the degree and variability of PTM in control and OI collagen support future molecular modeling studies to uncover how PTM affects the structure and packing of the collagen-I fibril.

We identified significant differences in the degree of PTM of collagen-I derived from bone, dentine and fibroblasts in OI. This suggests that factors beyond the intrinsic delayed folding of collagen-I have an effect on the PTMs levels. Collagen-I is part of distinct structures in different tissues. In bone, collagen-I fibrils form a foundation for a complex hierarchical organization resulting in a composite material, which is constantly remodeled through an intricate crosstalk between osteoblasts and osteoclasts (Garnero, 2015; Kim et al., 2020). In OI, bone is the site of the main clinical presentation. Dentine is avascular and non-remodeling. Dentinogenesis imperfecta occurs in 50 % of OI patients, leading to various dental issues such as discolored enamel, missing teeth, unerupted teeth, and malocclusion (Taqi et al., 2021a; Taqi et al., 2021b). The skin extracellular matrix is a fibrous network with lower matrix turnover (Mayorca-Guiliani et al., 2025). Individuals with OI do not report clinical issues related to skin, despite having mild alterations to skin elasticity (Hansen and Jemec, 2002). It is possible that tissue-specific environments require different levels of collagen-I PTM to allow for building structures that have vastly different functions (Bank et al., 1999; Eyre et al., 2019). If osteoblasts and fibroblasts have different levels of PTM enzymes or substrates, then delayed triple helix formation can lead to higher levels of over-modification in cells with higher levels of LH. Alternatively, the delay in folding of the triple helix may also be cell-type specific. It also should be noted that collagen from fibroblast is extracted after the cell culture step, whereas bone or dentine collagen is extracted directly using chemical treatment. To date, there have been no comparative studies on PTM protein gene expression and protein quantity across different tissues. Our analysis suggests that a high degree of PTM in OI bone collagen-I may explain why bone is so much more affected than other collagen-I containing tissues in OI.

Structural alterations due to high PTM in collagen-I likely contribute to bone fragility in OI. The increase in glycosylation, presented as the addition of two sugars residues along collagen-I would lead to a significant structural alteration compared to its healthy counterpart (Tvaroška, 2024; Schegg et al., 2009). In silico modeling of single tropocollagen demonstrated that GGHYL reduces the interfacial energy between collagen-I and the surrounding medium (Tang et al., 2020). The increased hydroxylation was shown to alter the cross-linking patterns, resulting in stiffer collagen-I fibrils (Lin and Gu, 2015). These molecular defects disrupt higher-order structures, likely leading to kinked, poorly packed tropocollagen and altered fibril diameter (Cassella et al., 1994). The hierarchical organization of bone amplifies structural defects of collagen-I fibrils, negatively affecting the mechanical properties of the entire bone matrix (Alcântara et al., 2022). The overall pathology of OI is complex and includes abnormal collagen-I structure, decreased collagen-I secretion, changes in cross-linking patterns, hyper-mineralized bone matrix, lower bone mineral density and higher osteoclast activity (Nijhuis et al., 2019; Tauer et al., 2019; Imbert et al., 2014). This complexity needs a multiscale approach to disease understanding, from the molecular level to the clinical presentation.

This study has several limitations. First, many of the included publications date back to a time when diagnostic tools for OI were less developed and less prevalent in the medical space. As a result, disease classification may have been inconsistent, potentially affecting the severity analysis. Additionally, non-collagen-I mutation forms of OI were not well-characterized until more recently, which may have led to improper inclusion of some studies. Even when genetic diagnosis was performed, the patient's genotype (or even if it was a base change mutation or a frameshift mutation) was severely under-reported, preventing genotype-PTM association studies. Another major limitation was the frequent lack of demographic information, such as age and sex, preventing exploration of these variables as potential modifiers of PTM levels. The studies that report age were skewed toward pediatric populations, pointing out a flaw in the research field of OI. Finally, the sample size for certain outcomes remains small; most notably, common glycosylation outcome data were available for only 17 patients across five studies. Expanding the number of studies and patients would help solidify trends that are not observable with the current sample size.

Our systematic review and meta-analysis provide the most comprehensive synthesis to date of biological data on collagen-I PTMs in individuals with OI due to collagen-I mutations. By combining data from 269 patients, we achieved a substantial sample size for this rare disease, providing novel insights into the pathophysiology of OI. Our findings provide a foundation for future work investigating the mechanistic link between collagen-I biochemistry and skeletal fragility in OI and have several translational implications. PTM profiles could be developed into biomarkers for early diagnosis, disease stratification, and monitoring of therapeutic response. They may also inform the design of targeted therapies that modulate collagen-I modification pathways, as well as the development of tissue-specific interventions and biomaterials for bone repair and regeneration. By providing a unified framework and benchmark dataset, this study bridges fundamental biology and clinical practice by supporting a multi-scale approach to understand OI pathophysiology, and laying the groundwork for integrating molecular data into personalized care strategies.

## Authors contributions

PP developed the search strategy, performed screening and data extraction, participated in development of data analysis protocols and performed the data analysis, wrote the first draft of the manuscript. SA performed screening, participated in development of data analysis protocols and edited the manuscript. SVK conceived the study, obtained funding, developed the data analysis protocols, participated in data analysis and edited the manuscript. All authors read and approved the final manuscript.

## CRediT authorship contribution statement

**Priyesh Patel:** Writing – review & editing, Writing – original draft, Visualization, Validation, Software, Methodology, Formal analysis, Data curation, Conceptualization. **Sirion Aksornthong:** Writing – review & editing, Validation, Data curation. **Svetlana V. Komarova:** Writing – review & editing, Supervision, Project administration, Formal analysis, Conceptualization.

## Funding

This work was supported by the Natural Sciences and Engineering Research Council (NSERC, RGPIN-288253) and Developmental Grant DGBR87210CAN24 from the Shriners of North America.

## Declaration of competing interest

The authors declare that they have no competing interests.

## Data Availability

Data will be made available on request.

## Appendix A Supplementary data

Supplementary material 1 Image 1

Supplementary material 2 Image 2

Supplementary material 3 Image 3
