# Supplementary material for: Post-translational modifications of collagen type I in osteogenesis imperfecta: Systematic review and meta-analysis
Source: Bone Rep. 2025 Dec 12;28:101894. doi: 10.1016/j.bonr.2025.101894 (PMC12794236; doi:10.1016/j.bonr.2025.101894)
Supplement: Supplementary file 1 — Supplementary material 1 [file mmc1.pdf]

Supplementary material for the manuscript “Post-translational modifications of collagen type I in osteogenesis imperfecta: systematic review and meta-analysis” by P. Patel, S. Aksornthong, and SV Komarova

Supplementary Method 1: Search Strategy

Example of search strategy applied for web of Science database

1 = (TS = (osteogenesis imperfecta))

2 = (TS = (brittle bone disease))

3 = (TS = (brittle bone dysplasia))

4 = (TS = (collagen))

5 = (TS = (biochemical properties))

6 = (TS = (Protein processing, Post-translational, hydroxylation))

7 = (TS = (lysine, hydroxylysine, lysine modification))

8 = (TS = (proline, hydroxyproline, proline modification))

9 = (TS = (glycosylation, galactosyltransferase,))

10 = (TS = (glucose, galactose, monosaccharide, disaccharide))

11 = #1 OR #2 OR #3

12 = #4 OR #5 OR #6 OR #7 OR #8 OR #9 OR #10

13 = #11 OR #12

Supplementary Method 2: Quality checklist

S2.1 Description of the checklist for quality assessment for clinical study

1. Was the OI type specified. (0.5 given for qualitative description, 1pts where type 2 can be inferred by perinatal lethal description, 1.5pts for type description based off silence classification, 2pts for type description and mutation)
2. Age description of patient (1 pts)
3. Sex description of patient (1 pts)
4. Did the study have age-matched control (1pts)
5. Was the experimental method described (1 pts)
6. Did they run triplicates (0.5 for duplicates, 1 pts for triplicates)
7. Was the method used the standard method (1 pts)
8. Did the patient pool have different types of OI (1pts)
9. Is the sample size larger than 5 (1 pts)
10. Does the study provide individual patient data (1pts)

Supplementary Table 1. PRISMA checklist

Section and Topic	Item #	Checklist item	Location where item is reported
**TITLE**			
Title	1	Identify the report as a systematic review.	1
**ABSTRACT**			
Abstract	2	See the PRISMA 2020 for Abstracts checklist.	Material and Methods paragraph 1
**INTRODUCTION**			
Rationale	3	Describe the rationale for the review in the context of existing knowledge.	Introduction paragraph 2
Objectives	4	Provide an explicit statement of the objective(s) or question(s) the review addresses.	Introduction paragraph 3
**METHODS**			
Eligibility criteria	5	Specify the inclusion and exclusion criteria for the review and how studies were grouped for the syntheses.	Material and Methods paragraph 1
Information sources	6	Specify all databases, registers, websites, organisations, reference lists and other sources searched or consulted to identify studies. Specify the date when each source was last searched or consulted.	Material and Methods paragraph 1
Search strategy	7	Present the full search strategies for all databases, registers and websites, including any filters and limits used.	Material and Methods paragraph 1
Selection process	8	Specify the methods used to decide whether a study met the inclusion criteria of the review, including how many reviewers screened each record and each report retrieved, whether they worked independently, and if applicable, details of automation tools used in the process.	Material and Methods paragraph 1
Data collection process	9	Specify the methods used to collect data from reports, including how many reviewers collected data from each report, whether they worked independently, any processes for obtaining or confirming data from study investigators, and if applicable, details of automation tools used in the process.	Material and Methods paragraph 2
Data items	10a	List and define all outcomes for which data were sought. Specify whether all results that were compatible with each outcome domain in each study were sought (e.g. for all measures, time points, analyses), and if not, the methods used to decide which results to collect.	Material and Methods paragraph 3
	10b	List and define all other variables for which data were sought (e.g. participant and intervention characteristics, funding sources). Describe any assumptions made about any missing or unclear information.	Material and Methods paragraph 1
Study risk of bias assessment	11	Specify the methods used to assess risk of bias in the included studies, including details of the tool(s) used, how many reviewers assessed each study and whether they worked independently, and if applicable, details of automation tools used in the process.	Material and Methods paragraph 1
Effect measures	12	Specify for each outcome the effect measure(s) (e.g. risk ratio, mean difference) used in the synthesis or presentation of results.	Material and Methods paragraph 4
Synthesis methods	13a	Describe the processes used to decide which studies were eligible for each synthesis (e.g. tabulating the study intervention characteristics and comparing against the planned groups for each synthesis (item #5)).	Material and Methods paragraph 1

Section and Topic	Item #	Checklist item	Location where item is reported
	13b	Describe any methods required to prepare the data for presentation or synthesis, such as handling of missing summary statistics, or data conversions.	Material and Methods paragraph 3
	13c	Describe any methods used to tabulate or visually display results of individual studies and syntheses.	Material and Methods paragraph 5
	13d	Describe any methods used to synthesize results and provide a rationale for the choice(s). If meta-analysis was performed, describe the model(s), method(s) to identify the presence and extent of statistical heterogeneity, and software package(s) used.	Material and Methods paragraph 5
	13e	Describe any methods used to explore possible causes of heterogeneity among study results (e.g. subgroup analysis, meta-regression).	Material and Methods paragraph 5
	13f	Describe any sensitivity analyses conducted to assess robustness of the synthesized results.	Material and Methods paragraph 6
Reporting bias assessment	14	Describe any methods used to assess risk of bias due to missing results in a synthesis (arising from reporting biases).	Material and Methods paragraph 6
Certainty assessment	15	Describe any methods used to assess certainty (or confidence) in the body of evidence for an outcome.	Material and Methods paragraph 6
**RESULTS**			
Study selection	16a	Describe the results of the search and selection process, from the number of records identified in the search to the number of studies included in the review, ideally using a flow diagram.	Figure 1
	16b	Cite studies that might appear to meet the inclusion criteria, but which were excluded, and explain why they were excluded.	
Study characteristics	17	Cite each included study and present its characteristics.	T3 and supplementary T2
Risk of bias in studies	18	Present assessments of risk of bias for each included study.	T3
Results of individual studies	19	For all outcomes, present, for each study: (a) summary statistics for each group (where appropriate) and (b) an effect estimate and its precision (e.g. confidence/credible interval), ideally using structured tables or plots.	Fig 2,3,4
Results of syntheses	20a	For each synthesis, briefly summarise the characteristics and risk of bias among contributing studies.	Supplementary Fig 2
	20b	Present results of all statistical syntheses conducted. If meta-analysis was done, present for each the summary estimate and its precision (e.g. confidence/credible interval) and measures of statistical heterogeneity. If comparing groups, describe the direction of the effect.	Fig 2,3,4
	20c	Present results of all investigations of possible causes of heterogeneity among study results.	Supplementary Fig 2
	20d	Present results of all sensitivity analyses conducted to assess the robustness of the synthesized results.	Supplementary Fig 2
Reporting biases	21	Present assessments of risk of bias due to missing results (arising from reporting biases) for each synthesis assessed.	Supplementary Fig 2
Certainty of evidence	22	Present assessments of certainty (or confidence) in the body of evidence for each outcome assessed.	Supplementary Fig 2
**DISCUSSION**			
Discussion	23a	Provide a general interpretation of the results in the context of other evidence.	Discussion paragraph 2,3

Section and Topic	Item #	Checklist item	Location where item is reported
	23b	Discuss any limitations of the evidence included in the review.	Discussion paragraph 4
	23c	Discuss any limitations of the review processes used.	Discussion paragraph 4 and Supplementary paragraph 1
	23d	Discuss implications of the results for practice, policy, and future research.	Discussion paragraph 5
**OTHER INFORMATION**			
Registration and protocol	24a	Provide registration information for the review, including register name and registration number, or state that the review was not registered.	Material and Methods paragraph 1
	24b	Indicate where the review protocol can be accessed, or state that a protocol was not prepared.	Not applicable
	24c	Describe and explain any amendments to information provided at registration or in the protocol.	Not applicable
Support	25	Describe sources of financial or non-financial support for the review, and the role of the funders or sponsors in the review.	Found in the “Funding” section of the publisher page
Competing interests	26	Declare any competing interests of review authors.	Found in the “Declaration of competing interest” section of the publisher page
Availability of data, code and other materials	27	Report which of the following are publicly available and where they can be found: template data collection forms; data extracted from included studies; data used for all analyses; analytic code; any other materials used in the review.	Patient characteristics are available in the supplementary. Raw data available upon request

Supplementary Table 2. Units of outcomes for HYL and HYP outcomes

Author and Title	Outcomes	Units
**Bleckmann, 1971**	HYL, HYP	Amino Acid Count
**Eastoe, 1973**	HYL, HYP	Amino Acid Count
**Meigel, 1975**	HYP	Ratio Pro/HYP
**Trelstad, 1977**	HYL	Amino Acid Count
**Kirsch, 1981**	HYL, HYP	Amino Acid Count and Percentage Hydroxylation,
**Herbage, 1982**	HYL	Amino Acid Count and HYL/HYP Ratio
**Kirsch, 1983**	HYL, HYP	Amino Acid Count by CNBr Peptides
**Steinmann, 1984**	HYL, HYP	Percentage Hydroxylation
**Deak, 1985**	HYL, HYP	Amino Acid Count, Percentage Hydroxylation
**Stoss, 1986**	HYL, HYP	Amino Acid Count per Collagen Chain, Percentage Hydroxylation
**Steinmann, 1986**	HYL, HYP	Percentage Hydroxylation per Collagen Chain
**Kirsch, 1987**	HYL	Percentage Hydroxylation
**Tenni, 1988**	HYL	Percentage Hydroxylation
**Brenner, 1988**	HYL	Ratio HYL/HYP
**Maroteaux, 1988**	HYL, HYP	Percentage Hydroxylation
**Rao, 1989**	HYL	Percentage Hydroxylation
**Brenner, 1989**	HYL	Ratio HYL/HYP
**Brenner, 1993**	HYL, HYP	Ratio HYL/HYP
**Tajima, 1994**	HYL, HYP	Ratio HYL/LYS and HYP/PRO
**Lehmann, 1995**	HYL, HYP	Percentage Hydroxylation
**Bank, 2000**	HYP	Ratio HYP/PRO
**Barnes, 2006**	HYL	Percentage Hydroxylation
**Makareeva, 2018**	HYL	Percentage Hydroxylation

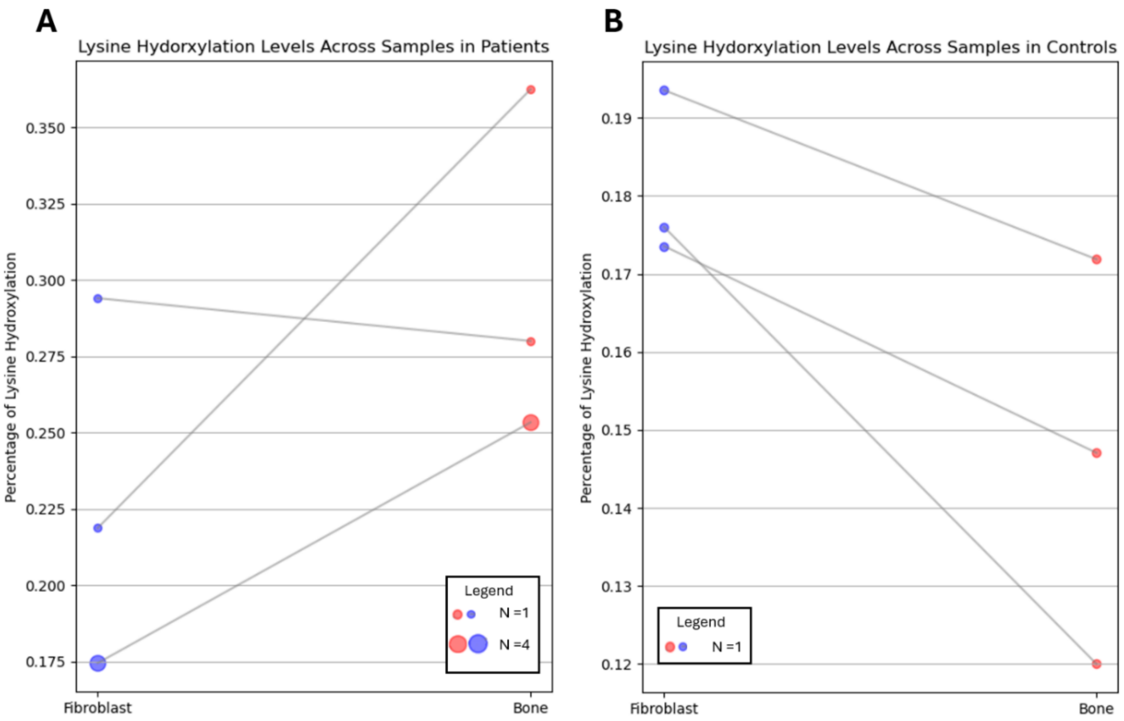

Supplementary Figure S1. Comparison of HYL levels in fibroblast- and bone-derived collagen. Data from three publications : Eastoe 1973, Krish 1981 and Herbage 1982 reporting HYL levels in both fibroblast- and bone-derived collagen from the same OI patients (A) and control (B). Lines connect data from the same individuals.

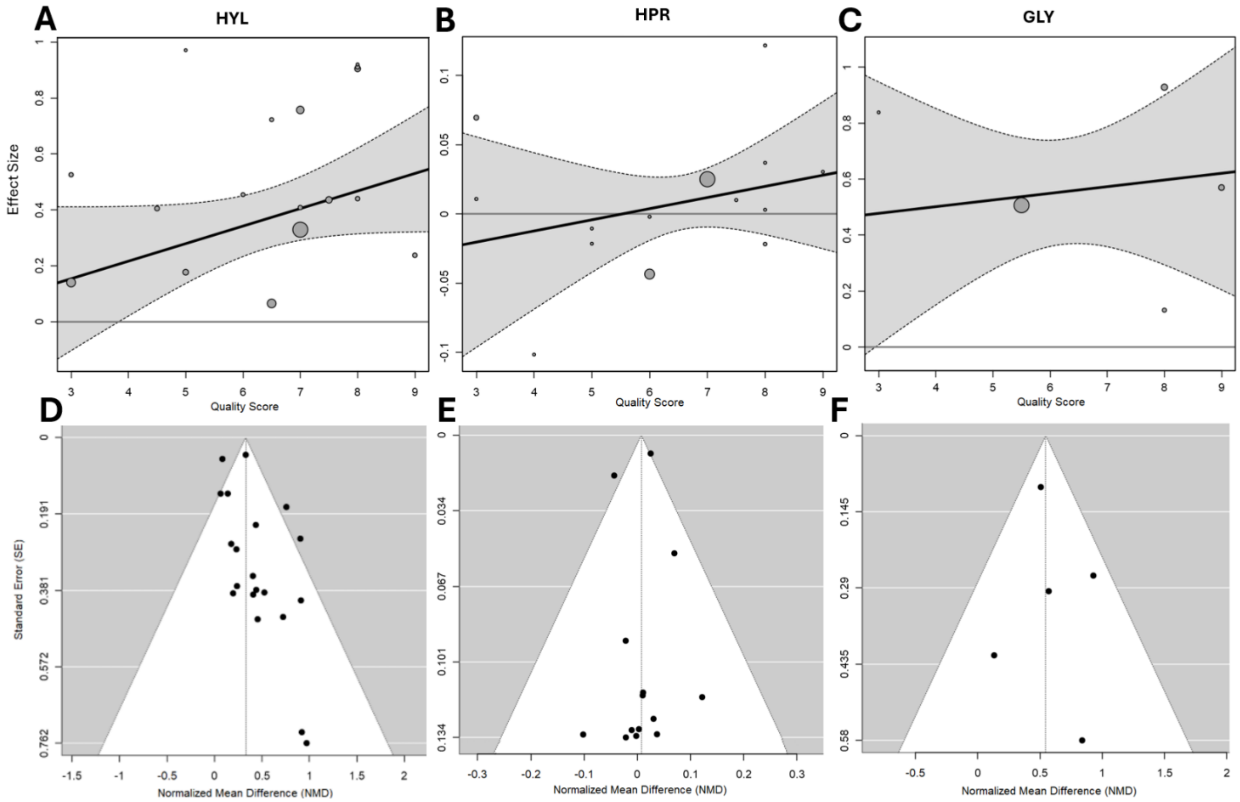

Supplementary Figure S2. Publication Bias Analysis. (A-C) Funnel plot for percentage change in HYL (A), percentage change in HYP (B), and percentage in change in glycosylation (C). (D-F) Association of the effect size with quality score for HYL (D), HYP (E) and glycosylation (F). The analysis was performed using datasets reported on figures 2A (HYL), 3A (HYP), and 4A (glycosylation).

Rational of Meta-analysis

This meta-analysis is largely composed of single-patient case studies. While such studies are typically excluded in conventional meta-analyses due to bias concerns, for rare diseases, this would eliminate a substantial portion of the available patient data. We have included these studies because the nature of the included studies focusing solely on biological measurements without any interventions and the use of invasive methodologies that inherently limit sample sizes, this make the potential for negative effects of bias less of a concern.

To address the absence of variance in single-patient case studies, we pooled all such studies to calculate a common standard deviation, which was then imputed across them. While we considered the possibility that measurement methods might influence the results, all studies used common methods depending on the sample the collagen is derived from. There are assumption that need to be taken when imputing data in this manner. The pooled standard deviation calculated for patients and control group results in a larger SE compared to studies with large patient groups, which is a conservative approach as it will result in under-weighting of smaller studies (each case study gets down-weighted). Heterogeneity statistics like Q and I^2 depend on the balance between within-study variance and between-study differences. Giving each one-person study a fairly large within-study variance will force I^2 to be very low. All outcomes in this meta-analysis had I^2 lower than 16.6% within subgroup. In contrast, τau^2 is insensitive to the precision of the studies, thus not affected by the imputation method. The τau^2 calculated in this meta-analysis is all very low because the individual effect sizes are very close to each other. The random effect model chosen is the Hunter-Schmidt estimator with small sample size correction, which down weight small sample size studies.
