# Supplementary material for: Post-translational modifications of collagen type I in osteogenesis imperfecta: Systematic review and meta-analysis
Source: Bone Rep. 2025 Dec 12;28:101894. doi: 10.1016/j.bonr.2025.101894 (PMC12794236; doi:10.1016/j.bonr.2025.101894)
Supplement: Supplementary file 2 — Supplementary material 2 [file mmc2.pdf]

Qualitative

1. Barsh GS, Byers PH. Reduced secretion of structurally abnormal type I procollagen in a form of osteogenesis imperfecta. *Proceedings of the National Academy of Sciences of the United States of America*. Aug 1981;78(8):5142-6. doi:https://doi.org/10.1073/pnas.78.8.5142
2. Sippola M, Kaffe S, Prockop DJ. A heterozygous defect for structurally altered pro-alpha 2 chain of type I procollagen in a mild variant of osteogenesis imperfecta. The altered structure decreases the thermal stability of procollagen and makes it resistant to procollagen N-proteinase. *The Journal of biological chemistry*. 1984 1984;259(22):14094-100. doi:https://doi.org/10.1016/S0021-9258(18)89861-1
3. De Vries WN, de Wet WJ. The molecular defect in an autosomal dominant form of osteogenesis imperfecta. Synthesis of type I procollagen containing cysteine in the triple-helical domain of pro-alpha 1(I) chains. *Journal of Biological Chemistry*. Jul 05 1986;261(19):9056-64. doi:https://doi.org/10.1016/S0021-9258(19)84487-3
4. Wenstrup RJ, Tsipouras P, Byers PH. Osteogenesis imperfecta type IV. Biochemical confirmation of genetic linkage to the pro alpha 2(I) gene of type I collagen. *Journal of Clinical Investigation*. Dec 1986;78(6):1449-55. doi:https://doi.org/10.1172/JCI112735
5. Wenstrup RJ, Hunter AG, Byers PH. Osteogenesis imperfecta type IV: evidence of abnormal triple helical structure of type I collagen. *Hum Genet*. Sep 1986;74(1):47-53. doi:https://doi.org/10.1007/BF00278784
6. Vogel BE, Minor RR, Freund M, Prockop DJ. A point mutation in a type I procollagen gene converts glycine 748 of the alpha 1 chain to cysteine and destabilizes the triple helix in a lethal variant of osteogenesis imperfecta. *Journal of Biological Chemistry*. Oct 25 1987;262(30):14737-44. doi:https://doi.org/10.1016/S0021-9258(19)84475-7
7. Bateman JF, Mascara T, Chan D, Cole WG. A structural mutation of the collagen alpha 1(I)CB7 peptide in lethal perinatal osteogenesis imperfecta. *Journal of Biological Chemistry*. Apr 05 1987;262(10):4445-51. doi:https://doi.org/10.1016/S0021-9258(18)61213-X
8. Bateman JF, Chan D, Walker ID, Rogers JG, Cole WG. Lethal perinatal osteogenesis imperfecta due to the substitution of arginine for glycine at residue 391 of the alpha 1(I) chain of type I collagen. *Journal of Biological Chemistry*. May 25 1987;262(15):7021-7. doi:https://doi.org/10.1016/S0021-9258(18)48196-3
9. Willing MC, Cohn DH, Starman B, Holbrook KA, Greenberg CR, Byers PH. Heterozygosity for a large deletion in the alpha 2(I) collagen gene has a dramatic effect on type I collagen secretion and produces perinatal lethal osteogenesis imperfecta. *Journal of Biological Chemistry*. Jun 15 1988;263(17):8398-404. doi:https://doi.org/10.1016/S0021-9258(18)68491-1
10. Byers PH, Starman BJ, Cohn DH, Horwitz AL. A novel mutation causes a perinatal lethal form of osteogenesis imperfecta. An insertion in one alpha 1(I) collagen allele (COL1A1). *Journal of Biological Chemistry*. Jun 05 1988;263(16):7855-61. doi:https://doi.org/10.1016/S0021-9258(18)68576-X
11. Tenni R, Cetta G, Dyne K, Valli M, Zanaboni G, Castellani AA. Severe nonlethal osteogenesis imperfecta: biochemical heterogeneity. *Annals of the New York Academy of Sciences*. 1988;543:73-82. doi:https://doi.org/10.1111/j.1749-6632.1988.tb55317.x
12. Bateman JFC, D. Lamande, Mascara, T. Cole, W. G. Biochemical Heterogeneity of type I collagen mutation in osteogenesis imperfecta. *Annals of the New York Academy of Sciences*. 1988;doi:https://doi.org/10.1111/j.1749-6632.1988.tb55321.x
13. Superti-Furga A, Royce PM, Pistone FM, Romano C, Steinmann B. Delayed triple-helix formation of abnormal type I collagen is corrected by reduced temperature. Studies of a family with variable

expression of osteogenesis imperfecta. *Annals of the New York Academy of Sciences*. 1988 1988;543:85-92. doi:https://doi.org/10.1111/j.1749-6632.1988.tb55319.x
14. Wenstrup RJ, Cohn DH, Cohen T, Byers PH. Arginine for glycine substitution in the triple-helical domain of the products of one alpha2(I) collagen allele (COL1A2) produces the osteogenesis imperfecta type IV phenotype. *Journal of Biological Chemistry*. 1988 1988;263(16):7734-7740. doi:https://doi.org/10.1016/S0021-9258(18)68560-6
15. Marini JC, Grange DK, Gottesman GS, Lewis MB, Koeplin DA. Osteogenesis imperfecta type IV. Detection of a point mutation in one alpha 1(I) collagen allele (COL1A1) by RNA/RNA hybrid analysis. *J Biol Chem*. Jul 15 1989;264(20):11893-900. doi:https://doi.org/10.1016/S0021-9258(18)80150-8
16. Constantinos D. Constantinou KBN, *, Prockop DJ. A lethal variant of osteogenesis imperfecta has a single base mutation that substitutes cysteine for glycine 904 of the alpha 1(I) chain of type I procollagen. The asymptomatic mother has an unidentified mutation producing an overmodified and unstable type I procollagen. *J Clin Invest*. 1989;doi:https://doi.org/10.1073/pnas.83.16.6045
17. Baldwin CT, Constantinou CD, Dumars KW, Prockop DJ. A single base mutation that converts glycine 907 of the alpha 2(I) chain of type I procollagen to aspartate in a lethal variant of osteogenesis imperfecta. The single amino acid substitution near the carboxyl terminus destabilizes the whole triple helix. *The Journal of biological chemistry*. 1989 1989;264(5):3002-6. doi:https://doi.org/10.1016/S0021-9258(19)81713-1
18. Starman BJ, Eyre D, Charbonneau H, et al. Osteogenesis imperfecta. The position of substitution for glycine by cysteine in the triple helical domain of the Proalpha1(I) chains of type I collagen determines the clinical phenotype. *Journal of Clinical Investigation*. 1989 1989;84(4):1206-1214. doi:https://doi.org/10.1172/JCI114286
19. Wallis GA, Starman BJ, Schwartz MF, Byers PH. Substitution of arginine for glycine at position 847 in the triple-helical domain of the alpha 1 (I) chain of type I collagen produces lethal osteogenesis imperfecta. Molecules that contain one or two abnormal chains differ in stability and secretion. *Journal of Biological Chemistry*. Oct 25 1990;265(30):18628-33. doi:https://doi.org/10.1016/S0021-9258(17)44798-3
20. Grange DK, Lewis MB, Marini JC. Analysis of cultured chorionic villi in a case of osteogenesis imperfecta type II: Implications for prenatal diagnosis. *American Journal of Medical Genetics*. 1990 1990;36(2):258-264. doi:https://doi.org/10.1002/ajmg.1320360223
21. Valli M, Tenni R, Cetta G. Moderately Severe Osteogenesis Imperfecta: Biochemical Studies Showing Variable Defect Localization in the Triple-Helical Domain of Type I Collagen. *Matrix*. 1990/07/01/ 1990;10(3):200-205. doi:https://doi.org/10.1016/S0934-8832(11)80169-1
22. Bateman JF, Hannagan M, Chan D, Cole WG. Characterization of a type I collagen alpha 2(I) glycine-586 to valine substitution in osteogenesis imperfecta type IV. Detection of the mutation and prenatal diagnosis by a chemical cleavage method. *Biochem J*. Jun 15 1991;276(Pt 3):765-70. doi:https://doi.org/10.1042/bj2760765
23. Tenni R, Biglino P, Dyne K, et al. Phenotypic variability and abnormal type I collagen unstable at body temperature in a family with mild dominant osteogenesis imperfecta. *Journal of inherited metabolic disease*. 1991 1991;14(2):189-201. doi:https://doi.org/10.1007/BF01800591
24. Hawkins JR, Superti-Furga A, Steinmann B, Dalgleish R. A 9-base pair deletion in COL1A1 in a lethal variant of osteogenesis imperfecta. *The Journal of biological chemistry*. 1991 1991;266(33):22370-4.
25. Nicholls AC, Oliver J, Renouf DV, Keston M, Pope FM. Substitution of cysteine for glycine at residue 415 of one allele of the alpha 1(I) chain of type I procollagen in type III/IV osteogenesis imperfecta. *J Med Genet*. 1991;28(11):757-64. doi:https://dx.doi.org/10.1136/jmg.28.11.757

26. Wallis GA, Kadler KE, Starman BJ, Byers PH. A tripeptide deletion in the triple-helical domain of the pro alpha 1(I) chain of type I procollagen in a patient with lethal osteogenesis imperfecta does not alter cleavage of the molecule by N-proteinase. *Journal of Biological Chemistry*. Dec 15 1992;267(35):25529-34. doi:https://doi.org/10.1016/S0021-9258(19)74072-1
27. Bateman JF, Moeller I, Hannagan M, Chan D, Cole WG. Lethal perinatal osteogenesis imperfecta due to a type I collagen alpha 2(I) Gly to Arg substitution detected by chemical cleavage of an mRNA:cDNA sequence mismatch. *Human Mutation*. 1992;1(1):55-62. doi:https://doi.org/10.1002/humu.1380010109
28. Fertala A, Westerhausen A, Morris G, Rooney JE, Prockop DJ. Two cysteine substitutions in procollagen I: a glycine replacement near the N-terminus of alpha 1(I) chain causes lethal osteogenesis imperfecta and a glycine replacement in the alpha 2(I) chain markedly destabilizes the triple helix. *Biochem J*. Jan 01 1993;289(Pt 1):195-9. doi:https://doi.org/10.1042/bj2890195
29. Valli M, Sangalli A, Rossi A, et al. Osteogenesis imperfecta and type-I collagen mutations. A lethal variant caused by a Gly910-->Ala substitution in the alpha 1 (I) chain. *European Journal of Biochemistry*. Feb 01 1993;211(3):415-9. doi:https://doi.org/10.1111/j.1432-1033.1993.tb17565.x
30. Marini JC, Lewis MB, Wang Q, Chen KJ, Orrison BM. Serine for glycine substitutions in type I collagen in two cases of type IV osteogenesis imperfecta (OI). Additional evidence for a regional model of OI pathophysiology. *The Journal of biological chemistry*. 1993 1993;268(4):2667-73. doi:https://doi.org/10.1016/S0021-9258(18)53826-6
31. Lightfoot SJ, Atkinson MS, Murphy G, Byers PH, Kadler KE. Substitution of serine for glycine 883 in the triple helix of the pro alpha 1 (I) chain of type I procollagen produces osteogenesis imperfecta type IV and introduces a structural change in the triple helix that does not alter cleavage of the molecule by procollagen N-proteinase. *The Journal of biological chemistry*. 1994 1994;269(48):30352-7. doi:https://doi.org/10.1016/S0021-9258(18)43820-3
32. Sarafova AP, Choi H, Forlino A, et al. Three novel type I collagen mutations in osteogenesis imperfecta type IV probands are associated with discrepancies between electrophoretic migration of osteoblast and fibroblast collagen. *Human mutation*. 1998 1998;11(5):395-403. doi:https://doi.org/10.1002/(SICI)1098-1004(1998)11:5<395::AID-HUMU7>3.0.CO;2-4
33. Pace JM, Atkinson M, Willing MC, Wallis G, Byers PH. Deletions and duplications of Gly-Xaa-Yaa triplet repeats in the triple helical domains of type I collagen chains disrupt helix formation and result in several types of osteogenesis imperfecta. *Human Mutation*. 2001/10/01 2001;18(4):319-326. doi:https://doi.org/10.1002/humu.1193
34. Pace JM, Kuslich CD, Willing MC, Byers PH. Disruption of one intra-chain disulphide bond in the carboxyl-terminal propeptide of the proalpha1(I) chain of type I procollagen permits slow assembly and secretion of overmodified, but stable procollagen trimers and results in mild osteogenesis imperfecta. *J Med Genet*. Jul 2001;38(7):443-9. doi:https://doi.org/10.1136/jmg.38.7.443
35. Pace JM, Chitayat D, Atkinson M, Wilcox WR, Schwarze U, Byers PH. A single amino acid substitution (D1441Y) in the carboxyl-terminal propeptide of the proα1(I) chain of type I collagen results in a lethal variant of osteogenesis imperfecta with features of dense bone diseases. *J Med Genet*. 2002;39(1):23-29. doi:https://doi.org/10.1136/jmg.39.1.23
36. Cabral WA, Mertts MV, Makareeva E, et al. Type I collagen triplet duplication mutation in lethal osteogenesis imperfecta shifts register of alpha chains throughout the helix and disrupts incorporation of mutant helices into fibrils and extracellular matrix. *The Journal of biological chemistry*. 2003 2003;278(12):10006-12. doi:https://doi.org/10.1074/jbc.M212523200

Quantitative

37. Trelstad RL, Rubin D, Gross J. Osteogenesis imperfecta congenita: evidence for a generalized molecular disorder of collagen. *Lab Invest*. May 1977;36(5):501-8.
38. Kirsch E, Krieg T, Remberger K, Fendel H, Bruckner P, Muller PK. Disorder of collagen metabolism in a patient with osteogenesis imperfecta (lethal type): increased degree of hydroxylation of lysine in collagen types I and III. *European Journal of Clinical Investigation*. Feb 1981;11(1):39-47. doi:https://doi.org/10.1111/j.1365-2362.1981.tb01763.x
39. Bateman JF, Mascara T, Chan D, Cole WG. Abnormal type I collagen metabolism by cultured fibroblasts in lethal perinatal osteogenesis imperfecta. *Biochem J*. 1984;217(1):103. doi:https://doi.org/10.1042/bj2170103
7. Bateman JF, Mascara T, Chan D, Cole WG. A structural mutation of the collagen alpha 1(I)CB7 peptide in lethal perinatal osteogenesis imperfecta. *The Journal of biological chemistry*. 1987;262(10):4445-51. doi:https://doi.org/10.1016/S0021-9258(18)61213-X
41. Kirsch E, Krieg T, Nerlich A, et al. Compositional analysis of collagen from patients with diverse forms of osteogenesis imperfecta. *Calcif Tissue Int*. Jul 1987;41(1):11-7. doi:https://doi.org/10.1007/BF02555125
42. Tajima S, Takehana M, Azuma N. Production of overmodified type I procollagen in a case of osteogenesis imperfecta. Case Reports. *J Dermatol*. Apr 1994;21(4):219-22. doi:https://doi.org/10.1111/j.1346-8138.1994.tb01726.x
43. Bank RA, Tekoppele JM, Janus GJ, et al. Pyridinium cross-links in bone of patients with osteogenesis imperfecta: evidence of a normal intrafibrillar collagen packing. *J Bone Miner Res*. Jul 2000;15(7):1330-6. doi:https://doi.org/10.1359/jbmr.2000.15.7.1330
44. Taga Y, Kusubata M, Ogawa-Goto K, Hattori S. Site-specific Quantitative Analysis of Overglycosylation of Collagen in Osteogenesis Imperfecta Using Hydrazide Chemistry and SILAC. *Journal of Proteome Research*. 2013/05/03 2013;12(5):2225-2232. doi:https://doi.org/10.1021/pr400079d
45. Takeyari S, Kubota T, Ohata Y, et al. 4-phenylbutyric acid enhances the mineralization of osteogenesis imperfecta iPSC-derived osteoblasts. *Journal of Biological Chemistry*. Nov 05 2020;05:05. doi:https://dx.doi.org/10.1074/jbc.RA120.014709

Meta-Analysis

46. Bleckmann H, Kresse H, Wollensak J, Buddecke E. [Glycosaminoglycan and collagen analyses in osteogenesis imperfecta]. *Zeitschrift fur Kinderheilkunde*. 1971;110(1):74-84. doi:https://doi.org/10.1007/bf00446347
47. Eastoe JE, Martens P, Thomas NR. The amino-acid composition of human hard tissue collagens in osteogenesis imperfecta and dentinogenesis imperfecta. *Calcified Tissue Research*. 1973;12(2):91-100. doi:https://doi.org/10.1007/BF02013724
48. Meigel WN, Muller PK. Disturbance in the regulation of the type of collagen synthesized in a form of osteogenesis imperfecta. Review. *European Journal of Biochemistry*. Aug 01 1975;59(31):1255-64. Kollagenkrankheiten. doi:https://doi.org/10.1111/j.1432-1033.1975.tb02429.x
37. Trelstad RL, Rubin D, Gross J. Osteogenesis imperfecta congenita: evidence for a generalized molecular disorder of collagen. *Lab Invest*. May 1977;36(5):501-8.

49. Takagi Y, Koshiba H, Kimura O, Kuboki Y, Sasaki S. Dentinogenesis imperfecta: evidence of qualitative alteration in the organic dentin matrix. *Journal of oral pathology*. 1980 1980;9(4):201-9. doi:https://doi.org/10.1111/j.1600-0714.1980.tb00378.x
38. Kirsch E, Krieg T, Remberger K, Fendel H, Bruckner P, Muller PK. Disorder of collagen metabolism in a patient with osteogenesis imperfecta (lethal type): increased degree of hydroxylation of lysine in collagen types I and III. *European Journal of Clinical Investigation*. Feb 1981;11(1):39-47. doi:https://doi.org/10.1111/j.1365-2362.1981.tb01763.x
50. Herbage D, Borsali F, Buffevant C, Flandin F, Aguercif M. Composition, cross-linking and thermal stability of bone and skin collagens in patients with osteogenesis imperfecta. *Metabolic Bone Disease & Related Research*. 1982;4(2):95-101. doi:https://doi.org/10.1016/0221-8747(82)90022-4
51. Kirsch E, Glanville RW, Krieg T, Muller P. Analysis of cyanogen bromide peptides of type I collagen from a patient with lethal osteogenesis imperfecta. Research Support, Non-U.S. Gov't. *Biochem J*. Jun 01 1983;211(3):599-603. doi:https://doi.org/10.1042/bj2110599
52. Cetta G, De Luca G, Tenni R, Zanaboni G, Lenzi L, Castellani AA. Biochemical investigations of different forms of osteogenesis imperfecta. Evaluation of 44 cases. *Connective Tissue Research*. 1983;11(2-3):103-11. doi:https://doi.org/10.3109/03008208309004847
54. Steinmann B, Nicholls A, Pope FM. Clinical variability of osteogenesis imperfecta reflecting molecular heterogeneity: cysteine substitutions in the alpha 1(I) collagen chain producing lethal and mild forms. *J Biol Chem*. Jul 5 1986;261(19):8958-64. doi:https://doi.org/10.1016/S0021-9258(19)84475-7
55. Deak SB, van der Rest M, Prockop DJ. Altered helical structure of a homotrimer of alpha 1(I)chains synthesized by fibroblasts from a variant of osteogenesis imperfecta. *Coll Relat Res*. Sep 1985;5(4):305-13. doi:https://doi.org/10.1016/S0174-173X(85)80020-0
56. Stoss H, Pontz BF, Pesch HJ, Ott R. Heterogeneity of osteogenesis imperfecta. Biochemical and morphological findings in a case of type III according to Sillence. *Eur J Pediatr*. Apr 1986;145(1-2):34-9. doi:https://doi.org/10.1007/BF00441849
41. Kirsch E, Krieg T, Nerlich A, et al. Compositional analysis of collagen from patients with diverse forms of osteogenesis imperfecta. *Calcif Tissue Int*. Jul 1987;41(1):11-7. doi:https://doi.org/10.1007/BF02555125
57. Tenni R, Cetta G, Dyne K, et al. Type I procollagen in the severe non-lethal form of osteogenesis imperfecta. Defective pro-alpha 1(I) chains in a patient with abnormal proteoglycan metabolism and mineral deposits in the dermis. *Hum Genet*. Jul 1988;79(3):245-50. doi:https://doi.org/10.1007/BF00366245
58. Brenner RE, Vetter U, Worsdorfer O, Nerlich A, Teller WM, Muller PK. Biochemical analysis of bone compacta in osteogenesis imperfecta. *Annals of the New York Academy of Sciences*. 1988;543:106-8. doi:https://doi.org/10.1111/j.1749-6632.1988.tb55322.x
59. Maroteaux P, Cohen-Solal L, Bonaventure J. Clinical and genetical heterogeneity of osteogenesis imperfecta. Review. *Annals of the New York Academy of Sciences*. 1988;543:16-29. doi:https://doi.org/10.1111/j.1749-6632.1988.tb55312.x
60. Gage JP, Francis MJ, Smith R. Abnormal amino acid analyses obtained from osteogenesis imperfecta dentin. *Journal of dental research*. 1988 1988;67(8):1097-102. doi:https://doi.org/10.1177/00220345880670080701
61. Rao VH, Steinmann B, de Wet W, Hollister DW. Decreased thermal denaturation temperature of osteogenesis imperfecta mutant collagen is independent of post-translational overmodifications of lysine and hydroxylysine. *Journal of Biological Chemistry*. Jan 25 1989;264(3):1793-8. doi:https://doi.org/10.1016/S0021-9258(18)94257-2
62. Brenner RE, Vetter U, Nerlich A, Worsdorfer O, Teller WM, Muller PK. Biochemical analysis of callus tissue in osteogenesis imperfecta type IV. Evidence for transient overmodification in collagen

types I and III. *Journal of Clinical Investigation*. Sep 1989;84(3):915-21. doi:https://doi.org/10.1172/JCI114253
42. Tajima S, Takehana M, Azuma N. Production of overmodified type I procollagen in a case of osteogenesis imperfecta. Case Reports. *J Dermatol*. Apr 1994;21(4):219-22. doi:https://doi.org/10.1111/j.1346-8138.1994.tb01726.x
63. Brenner RE, Vetter U, Stoss H, Muller PK, Teller WM. Defective collagen fibril formation and mineralization in osteogenesis imperfecta with congenital joint contractures (Bruck syndrome). *Eur J Pediatr*. Jun 1993;152(6):505-8. doi:https://doi.org/10.1007/BF01955060
64. Lehmann HW, Rimek D, Bodo M, et al. Hydroxylation of collagen type I: evidence that both lysyl and prolyl residues are overhydroxylated in osteogenesis imperfecta. *European Journal of Clinical Investigation*. May 1995;25(5):306-10. doi:https://doi.org/10.1111/j.1365-2362.1995.tb01706.x
43. Bank RA, Tekoppele JM, Janus GJ, et al. Pyridinium cross-links in bone of patients with osteogenesis imperfecta: evidence of a normal intrafibrillar collagen packing. *J Bone Miner Res*. Jul 2000;15(7):1330-6. doi:https://doi.org/10.1359/jbmr.2000.15.7.1330
65. Barnes AM, Chang W, Morello R, et al. Deficiency of cartilage-associated protein in recessive lethal osteogenesis imperfecta. *N Engl J Med*. Dec 28 2006;355(26):2757-64. doi:https://doi.org/10.1056/NEJMoa063804

66. Makareeva E, Cabral WA, Marini JC, Leikin S. Molecular mechanism of alpha 1(I)-osteogenesis imperfecta/Ehlers-Danlos syndrome: unfolding of an N-anchor domain at the N-terminal end of the type I collagen triple helix. *The Journal of biological chemistry*. 2006 2006;281(10):6463-70. doi:https://doi.org/10.1074/jbc.M511830200
